# TET2 catalyzes active DNA demethylation of the *Sry* promoter and enhances its expression

**DOI:** 10.1038/s41598-019-50058-7

**Published:** 2019-09-17

**Authors:** Naoki Okashita, Shunsuke Kuroki, Ryo Maeda, Makoto Tachibana

**Affiliations:** 1 Division of Epigenome Dynamics, Institute of Advanced Medical Sciences, Tokushima University, 3-18-15 Kuramoto-Cho, Tokushima, 770-8503 Japan; 2 Laboratory of Epigenome Dynamics, Graduate School of Frontier Biosciences, Osaka University, 1-3 Yamadaoka, Suita, Osaka 565-0871 Japan

**Keywords:** DNA methylation, Developmental biology

## Abstract

SRY is the master regulator of male sex determination in eutherian mammals. In mice, *Sry* expression is transcriptionally and epigenetically controlled in a developmental stage-specific manner. The *Sry* promoter undergoes demethylation in embryonic gonadal somatic cells at the sex-determining period. However, its molecular mechanism and *in vivo* significance remain unclear. Here, we report that the *Sry* promoter is actively demethylated during gonadal development, and TET2 plays a fundamental role in *Sry* demethylation. *Tet*2-deficient mice showed absence of 5-hydroxymethylcytosine in the *Sry* promoter. Furthermore, *Tet2* deficiency diminished *Sry* expression, indicating that TET2-mediated DNA demethylation regulates *Sry* expression positively. We previously showed that the deficiency of the H3K9 demethylase *Jmjd1a* compromises *Sry* expression and induces male-to-female sex reversal. *Tet2* deficiency enhanced the sex reversal phenotype of *Jmjd1a*-deficient mice. Thus, TET2-mediated active DNA demethylation and JMJD1A-mediated H3K9 demethylation contribute synergistically to sex determination.

## Introduction

Expression of developmental genes is tuned through crosstalk between transcription factors and epigenetic regulation. The mammalian sex determining gene, *Sry*, is expressed in a certain population of somatic cells, termed as pre-Sertoli cells, in sexually undifferentiated embryonic gonads, thereby triggering the male development pathway^1–3^. *Sry* is expressed in a highly time-specific manner, *i*.*e*., its expression starts around embryonic day 10.5 (E10.5), peaks at around E11.5, and almost disappears by E12.5^4–8^. *Sry* expression is positively regulated by several transcription factors^9^. However, research on epigenetic mechanisms that contribute to *Sry* regulation is in its infancy. We previously reported that the H3K9 demethylase JMJD1A (also known as TSGA/JHDM2A/KDM3A) plays a pivotal role in mouse sex determination through *Sry* activation^10^. Recently, it was reported that histone acetyltransferases are also involved in *Sry* activation^11^.

In addition to histone modification, DNA methylation plays a pivotal role in developmental gene regulation^12,13^. DNA methylation is found to occur predominantly on cytosine followed by guanine residues (CpG)^14–16^. DNA methylation is induced by the *de novo* DNA methyltransferases DNMT3A/DNMT3B, and is maintained by a maintenance DNA methyltransferase DNMT1 during DNA replication. CpG methylation marks can be removed by replication-dependent and independent mechanisms^17^. The former is regulated by inhibition of DNA methyltransferase activity during *de novo* DNA synthesis, whereas the latter (also known as active demethylation) is induced by the oxidation of 5-methylcytosine (5mC) by ten-eleven translocation proteins (TET1/TET2/TET3) to produce 5-hydroxymethylcytosine (5hmC)^18^. 5hmC is further oxidized to 5-formylcytosine (5fC) and 5-carboxycytosine (5caC) by TET enzymes, both of which can be repaired by the base excision repair (BER) pathway to produce unmodified cytosine^19^. Previous studies have reported that the CpG sequences of the *Sry* promoter are demethylated in gonadal somatic cells at the sex-determining period^20,21^. These observations indicated that DNA demethylation in *Sry* promoter preceded *Sry* expression onset and that DNA demethylation was more pronounced in the *Sry* promoter region than in other *Sry* loci^20^. Furthermore, promoter activity assay showed that *in vitro* methylation of the 5′-flanking region of *Sry* suppressed reporter activity^21^. Although these results suggest a possible link between DNA demethylation and *Sry* expression, the regulatory mechanism of DNA demethylation in *Sry* promoter and its functional significance for sex determination remain elusive.

Here, we show that the active DNA demethylation pathway is involved in *Sry* regulation. 5hmC levels on *Sry* promoter were increased with increasing *Sry* expression in the somatic cells of developing gonads. Deficiency of *Tet2*, but not *Tet1*/*Tet*3, induced an increase in DNA methylation and disappearance of 5hmC in *Sry* promoter, indicating the pivotal role of TET2 in the dynamic regulation of DNA methylation in *Sry* promoter. Importantly, *Sry* expression was diminished in *Tet2*-deficient gonadal somatic cells at the sex-determining period. Furthermore, *Tet2* deficiency had a synergistic effect on the sex reversal phenotype, observed in a *Jmjd1a*-deficient background. These results identify TET2 as a responsible enzyme for DNA demethylation in *Sry* promoter and reveal that active DNA demethylation acts synergistically with histone modifications for epigenetic regulation of *Sry* and male sex determination.

## Results

### 5-hydroxymethylcytosine is preferentially enriched in NR5A1-positive gonadal somatic cells

**Figure 1:**
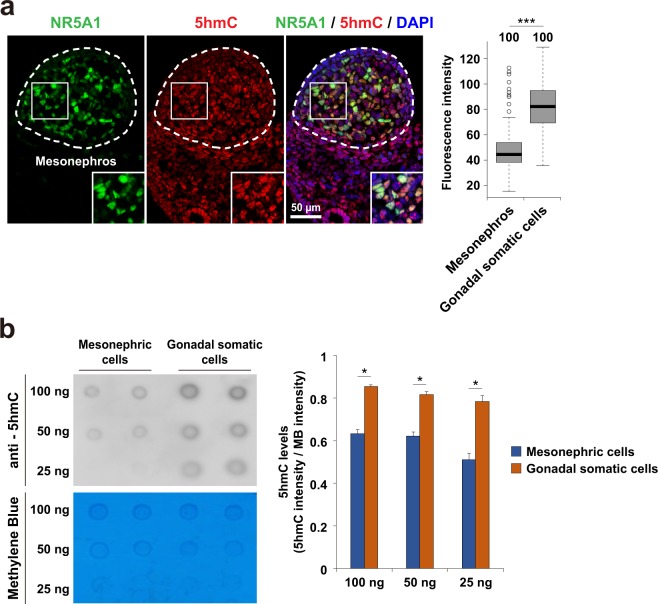
5-hydroxymethylcytosine is preferentially enriched in NR5A1-positive gonadal somatic cells. (**a**) Co-immunostaining profiles of NR5A1 and 5hmC in the central regions of XY E11.5 gonads. Enlarged boxes indicate co-localization of NR5A1 and 5hmC in gonadal somatic cells. Fluorescence intensity values of 5hmC in every 100 gonadal somatic cells and mesonephric cells were examined and summarized in a box plot (right). Signal intensity was quantified using ImageJ software. ***P < 0.001. n = 100. (**b**) Comparison of 5hmC amounts between mesonephric cells and gonadal somatic cells. Mesonephric cells and gonadal somatic cells were separated from XY embryos at E11.5 that carried *Nr5a1-hCD271-*transgene (see Materials and Method section) and were introduced into Dot-blot analysis. The relative amounts of 5hmC were calculated by dividing the signal intensity of 5hmC by that of methylene blue (right). Signal intensity was quantified using ImageJ software. Error bars indicate the SEM values of duplicates of the dot signal intensity. *P < 0.05.

Active DNA demethylation plays important roles in the processes of development and differentiation in mammals^22^. 5hmC, an intermediate in the active DNA demethylation pathway, is generated by oxidation of 5mC. To elucidate whether active DNA demethylation occurs during embryonic gonadal development, we performed double immunostaining analyses on XY embryonic gonad sections at the sex-determining period (E11.5) with antibodies against 5hmC and NR5A1 (also known as AD4BP/SF-1), which is transcription factor expressed in gonadal somatic cells but not in germ cells and mesonephric cells. We observed strong 5hmC signals in NR5A1-positive gonadal somatic cells, whereas these were weak in mesonephric cells (Fig. 1a, left). Quantitative analysis indicated that the average intensity of 5hmC was about two-fold higher in NR5A1-positive gonadal somatic cells compared to that in mesonephric cells (Fig. 1a, right). These data suggest that active DNA demethylation might arise in developing gonads around the sex-determining period.

To confirm the previous result, we measured global 5hmC levels in gonadal somatic cells and mesonephric cells by dot-blot analysis. We previously established *Nr5a1-hCD271*-transgenic (tg) mice in which NR5A1-positive gonadal somatic cells were tagged with a cell surface antigen, hCD271^10,23^. Using this tg line, we isolated NR5A1-positive cells (hereafter referred as gonadal somatic cells) from gonad-mesonephros pairs of E11.5 XY embryos, and then used them for 5hmC quantification. 5hmC levels in gonadal somatic cells were higher than those in mesonephric cells (Fig. 1b, left). Quantification analysis showed that 5hmC content was about 1.5-fold higher in gonadal somatic cells than that in mesonephric cells (Fig. 1b, right). These findings support the fact that active demethylation occurs preferentially in the gonadal somatic cell population at the sex-determining period.

### *Sry* promoter undergoes active DNA demethylation during gonadal development

**Figure 2:**
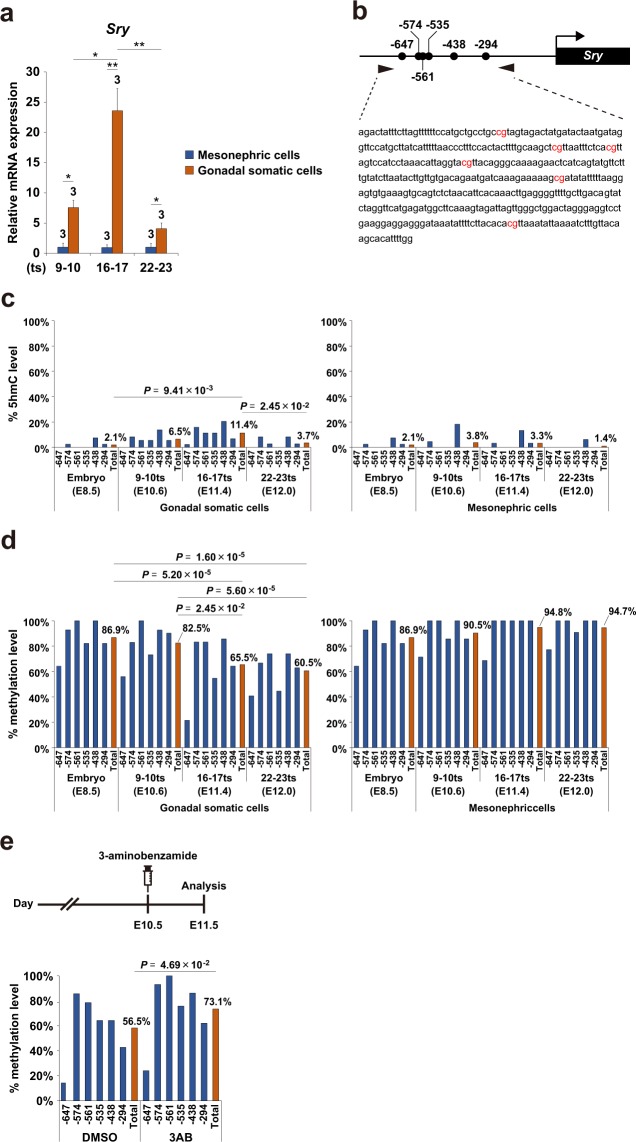
*Sry* promoter undergoes active DNA demethylation during gonadal development. (**a**) qRT-PCR analysis of *Sry* in XY mesonephric cells and gonadal somatic cells at the indicated ts stages. mRNA expression levels in mesonephric cells at the 9–10 ts stage (E10.6) were defined as 1. Data are presented as mean ± SD. *P < 0.05, **P < 0.01. n = 3. (**b**) Schematic representation of *Sry* promoter. *Sry* promoter contains 6 CpG sites. The positions of CpG sites are indicated relative to the start codon. (**c** and **d**) DNA methylation kinetics on *Sry* promoter in XY E8.5 embryos and XY mesonephric/gonadal somatic cells at the indicated developmental stages by Tet-assisted bisulfite sequencing analysis (**c**) and bisulfite sequencing analysis (**d**). Blue bars show the average percentage of methylation at individual CpG sites respectively and the red bar indicates that on total CpG sites. *P* values for bisulfite sequencing were obtained using the non-parametric two-tailed Mann-Whitney *U* test. (**e**) Administration of BER inhibitor inhibits DNA demethylation in the *Sry* promoter in developing gonads. The experimental scheme is shown in the upper panel. A BER inhibitor 3-aminobenzamide (3-AB) was intraperitoneally injected into pregnant females carrying E10.5 embryos. At 24 hours after injection, gonadal somatic cells were purified from XY embryos and used for bisulfite sequencing analysis. The lower panel shows DNA methylation levels of *Sry* promoter in 3-AB-treated and DMSO-treated (as vehicle control) XY gonadal somatic cells. The blue bar shows the average percentage of methylation at individual CpG sites and the red bar shows that of total CpG sites. *P* values for bisulfite sequencing were obtained using the non-parametric two-tailed Mann-Whitney *U* test.

To examine the kinetic relationship between *Sry* expression and DNA methylation/demethylation of *Sry*, we collected gonadal somatic cells from XY embryos at the tail somite (ts) stages 9 to 23 (E10.6-E12.0). In accordance with the known expression profile of *Sry*, its transcripts were highly enriched in the gonadal somatic cell fraction with a peak at the ts stage 16–17 (E11.4) (Fig. 2a). As shown in Fig. 2b, the *Sry* promoter contains 6 CpG sites. Genomic DNA isolated from gonadal somatic cells was used for Tet-assisted bisulfite (TAB) sequencing analysis, by which 5hmC can be quantitatively detected at single-base resolution^24^ (Fig. 2c). We found that 5hmC was detected in the *Sry* promoter in gonadal somatic cells, whereas it was barely detectable in E8.5 embryos and mesonephric cells (Fig. 2c). Notably, 5hmC levels in the *Sry* promoter in gonadal somatic cells fluctuated with kinetics similar to those of *Sry* expression during gonadal development (compare Fig. 2a with Fig. 2c). To confirm the correlation between 5hmC enrichment and DNA demethylation dynamics, we next examined DNA methylation (5mC + 5hmC) levels in the *Sry* promoter in gonadal somatic cells by bisulfite sequencing (Fig. 2d). With the development of gonads, DNA methylation levels of the *Sry* promoter were reduced progressively in gonadal somatic cells, whereas those of in E8.5 embryos and mesonephric cells were constantly close to 100% (Fig. 2d). These results collectively suggest that active DNA demethylation through 5mC oxidation might account for the reduction of DNA methylation in *Sry* promoter in the developing gonads, at least in part.

To confirm the involvement of the active DNA demethylation pathway in *Sry* regulation, we utilized 3-aminobenzamide (3-AB), a pharmacological inhibitor of the BER components PARP1. Several studies showed that 3-AB preserves 5mC levels by blocking the BER-dependent DNA demethylation pathway in primordial germ cells and embryonic stem cells^25–27^. 3-AB was intraperitoneally injected into pregnant females carrying E10.5 XY *Nr5a1-hCD271*-tg embryos (Fig. 2e). Gonadal somatic cells were isolated from the corresponding embryos 24 hours after injection and used for bisulfite sequence analysis (Fig. 2e). The averages of DNA methylation at the *Sry* promoter were 56.5% and 73.1% in the control and 3-AB treated gonadal somatic cells, respectively (Fig. 2e). The higher level of DNA methylation in 3-AB-treated cells supports the involvement of the active DNA demethylation pathway in the *Sry* regulation of gonadal somatic cells.

### TET2 plays a pivotal role in active DNA demethylation of the *Sry* promoter

**Figure 3:**
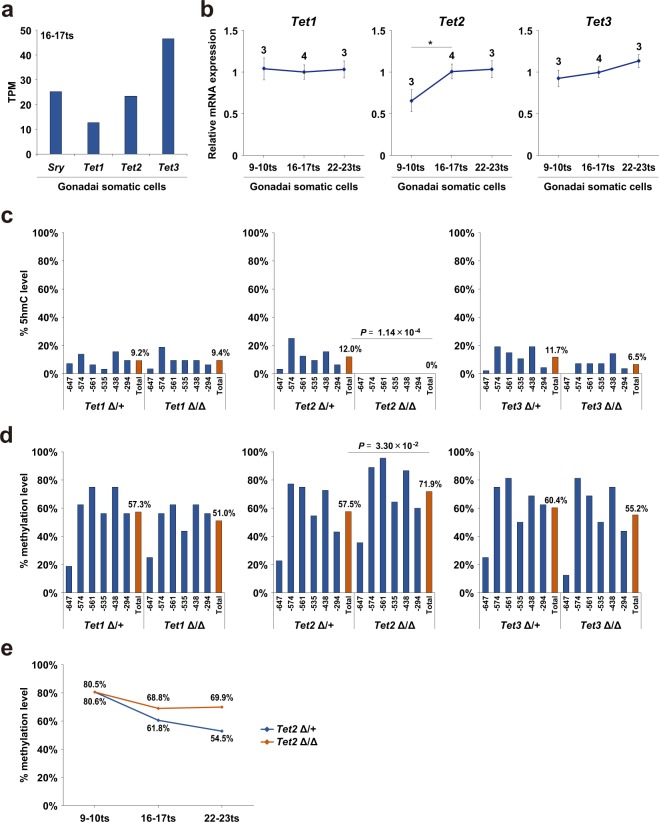
TET2 plays a pivotal role in active DNA demethylation of *Sry* promoter. (**a**) RNA-seq based gene expression values (TPM) of *Sry*, *Tet1*, *Tet2*, and *Tet3* in gonadal somatic cells at the sex-determining period. n = 2. (**b**) Expression kinetics of *Tet1*, *Tet2*, and *Tet3* in gonadal somatic cells. mRNAs were collected from XY gonadal somatic cells at the indicated ts stages and introduced into qRT-PCR analysis. mRNA expression levels in gonadal somatic cells at 16–17 ts stage were defined as 1. Data are presented as mean ± SD. * *P* < 0.05. n ≥ 3. (**c** and **d**) DNA methylation levels in the *Sry* promoter in E11.5 XY gonadal somatic cells of the indicated genotypes were measured by TAB sequencing (**c**) and bisulfite sequencing. (**d**) Blue bar shows the average percentage of methylation at individual CpG sites and red bar shows the average percentage of methylation on total CpG sites. *P* values for bisulfite sequencing were obtained using the non-parametric two-tailed Mann-Whitney *U* test. (**e**) Comparison of DNA methylation kinetics in the *Sry* promoter in develoing gonads. Gonadal somatic cells were purified from XY *Tet2*Δ/+ and *Tet2*Δ/Δ embryos at the indicated ts stages and were then used for bisulfite sequencing analysis.

Active DNA demethylation is triggered by hydroxylation of 5mC to 5hmC, which is catalyzed by TET proteins^18^. Because all Tet subfamily proteins, TET1, TET2, and TET3, have an enzymatic activity toward 5hmC production, we aimed to identify the enzyme responsible for *Sry* demethylation. mRNAs for *Tet1*, *Tet2*, and *Tet3* were detected in the gonadal somatic cells at the 16–17ts stage when *Sry* expression reached a peak (Fig. 3a and Supplementary Table S1). As shown in Fig. 3b, we found that mRNAs of *Tet2* were gradually increased, whereas those of *Tet1* and *Tet3* were unchanged in the somatic cells of developing gonads. To address the loss of function phenotype of TET enzymes, we generated mice carrying each mutant allele for *Tet1*, *Tet2*, and *Tet3* (hereafter described as *Tet1*Δ, *Tet2*Δ, and *Tet3*Δ) using the CRISPR/cas9 system (Supplementary Fig. S1). Each of the *Tet*-mutant lines was crossed with the *Nr5a1-hCD271*-tg line for further analysis. Gonadal somatic cells were immuno-magnetically collected from corresponding mutant embryos at the 16–17ts stage and used for TAB and bisulfite sequencing analysis (Fig. 3c,d, respectively). Strikingly, TAB sequence analysis demonstrated that 5hmC at the *Sry* promoter was completely abolished in *Tet2*Δ/Δ gonadal somatic cells, whereas it was unchanged in *Tet1*Δ/Δ or *Tet3*Δ/Δ cells (Fig. 3c). As shown in Fig. 3d, bisulfite sequencing analysis indicated that homozygous mutation of *Tet2* induced a significant increase in DNA methylation in the *Sry* promoter of gonadal somatic cells, whereas mutation of *Tet1* and *Tet3* did not (Fig. 3d). We next examined the kinetics of DNA methylation in *Sry* promoter during gonadal development from ts stages 9 to 23. As summarized in Fig. 3e, DNA methylation levels were progressively decreased in control cells but were almost unchanged in *Tet2*Δ/Δ gonadal somatic cells. From these results, we conclude that TET2 is the bona fide enzyme responsible for active demethylation at *Sry* promoter during gonadal development.

### *Tet2* mutation diminishes *Sry* expression in gonadal somatic cells and enhances sex-reversal in XY *Jmjd1a*Δ/Δ mice

**Figure 4:**
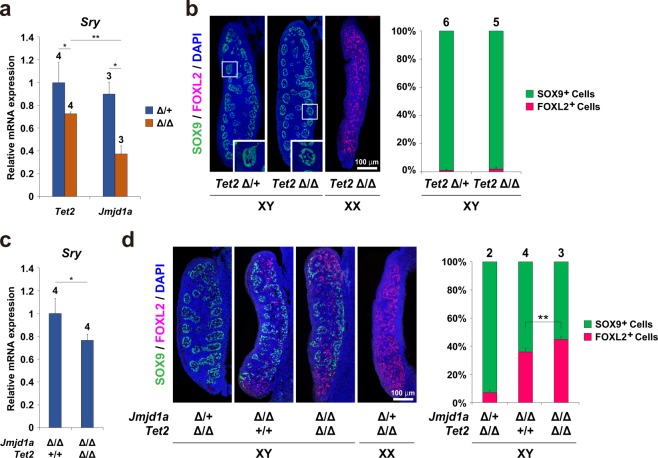
*Tet2* mutation diminishes *Sry* expression in gonadal somatic cells and enhances sex-reversal in XY *Jmjd1a*Δ/Δ mice. (**a**) Comparison of *Sry* mRNA levels between genotypes at the sex-determining period. Gonadal somatic cells were purified from E11.5 embryos of the indicated genotypes and were then used for qRT-PCR analysis. mRNA expression levels in *Tet2*Δ/+ gonadal somatic cells were defined as 1. Data are presented as mean ± SD. *P < 0.05, **P < 0.01. n ≥ 3. (**b**) Evaluation of sex development of *Tet2*-deficient E13.5 gonads by immunofluorescence analysis using antibodies against SOX9 and FOXL2. SOX9 and FOXL2 are markers for testicular Sertoli cells and ovarian somatic cells, respectively. The enlarged box demonstrates testicular tubule-like structures (left). The ratio of SOX9-positive cells to FOXL2-positive cells is summarized in the right. Numbers of embryos examined are shown above the bars. Data are presented as mean ± SD. (**c**) qRT-PCR analysis of *Sry* in XY *Jmjd1a*-deficient gonads and *Jmjd1a*/*Tet2*-deficient gonads at E11.5. Each of the samples included one pair of gonads/mesonephros. mRNA expression levels in *Jmjd1a*-deficient gonads were defined as 1. Data are presented as mean ± SD. *P < 0.05. n = 4. (**d**) Sex development of E13.5 embryonic gonads of the indicated genotypes was evaluated as in Fig. 4b. The ratio of SOX9-positive cells to FOXL2-positive cells is summarized in the right. Numbers of embryos examined are shown above the bars. Data are presented as mean ± SD. *P < 0.05.

We next aimed to address the role of TET2-mediated DNA demethylation in mouse sexual development. We first examined whether *Sry* expression was affected in *Tet2*Δ/Δ gonadal somatic cells at the sex-determining period. As shown in Fig. 4a, quantitative mRNA expression analysis indicated that *Sry* expression was significantly reduced in *Tet2*Δ/Δ gonadal somatic cells, indicating involvement of TET2-mediated DNA demethylation in regulating *Sry* expression. Our previous study demonstrated that JMJD1A-mediated H3K9 demethylation contributes to the regulation of *Sry* expression in developing gonads^26^. Comparative and quantitative analysis of *Sry* mRNA demonstrated that the decreased level of *Sry* expression by *Tet2*Δ/Δ mutation was moderate compared to that by *Jmjd1a* mutation (Fig. 4a). We next examined whether *Tet2* deficiency influences sexual development in embryonic gonads. For this, we performed co-immunostaining analysis on E13.5 gonad sections using antibodies against the testicular Sertoli cell marker SOX9 and the ovarian somatic cell marker FOXL2. As shown in Fig. 4b, *Tet2*Δ/Δ gonads possessed typical features of embryonic testis, in which SOX9-positive cells were abundant and testicular tubule formation proceeded, indicating that *Tet2* mutation alone does not induce sex reversal in embryonic gonads. It is possible that *Tet2* deficiency might affect DNA methylation levels of *Sox9* and *Foxl2*, and thereby influence this expression. To address this issue, we examined DNA methylation levels at the *Sox9* and *Foxl2* promoters in E11.5 gonadal somatic cells. As shown in Supplementary Fig. 2, *Tet2* deficiency did not alter the DNA methylation levels of *Sox9* and *Foxl2* promoters in gonadal somatic cells.

Our previous study demonstrated that *Jmjd1a* deficiency does not induce a significant increase in the DNA methylation of *Sry* promoter in gonadal somatic cells, suggesting that active DNA demethylation in *Sry* promoter occurs independently of JMJD1A-mediated H3K9 demethylation^28^. To address the synergistic effect between TET2-mediated DNA demethylation and JMJD1A-mediated H3K9 demethylation on gonadal sex development, we generated *Tet2*/*Jmjd1a*-double deficient mice and examined their gonadal sex development. *Sry* expression levels of *Tet2*/*Jmjd1a*-double deficient gonads were significantly lower than those of *Jmjd1a*-deficient gonads at the sex-determining period (Fig. 4c). *Jmjd1a*-deficient gonads at E13.5 were ovotestes composed of testicular cells and ovarian cells (Fig. 4d). Importantly, the ratio of FOXL2-positive cells to SOX9-positive cells was increased in *Tet2*/*Jmjd1a*-double deficient gonads as compared to *Jmjd1a*-deficient gonads (Fig. 4d). These results indicate that TET2-mediated DNA demethylation controls testicular development positively and synergistically with JMJD1A-mediated H3K9 demethylation.

## Discussion

The CpG sites of *Sry* promoter are demethylated in gonadal somatic cells at the sex-determining period^20,21^. However, its molecular mechanism and *in vivo* significance remain elusive. Here, we discovered that 5hmC was highly enriched in XY gonadal somatic cells at the sex-determining period and that the 5hmC level was increased in *Sry* promoter concomitantly with *Sry* expression in these cells. We also identified TET2 as the enzyme responsible for active DNA demethylation in *Sry* promoter. Finally, we revealed that TET2-mediated active DNA demethylation is involved in regulating *Sry* expression and sex development. Here we discuss the role of DNA demethylation in the epigenetic regulation of *Sry*.

**Figure 5:**
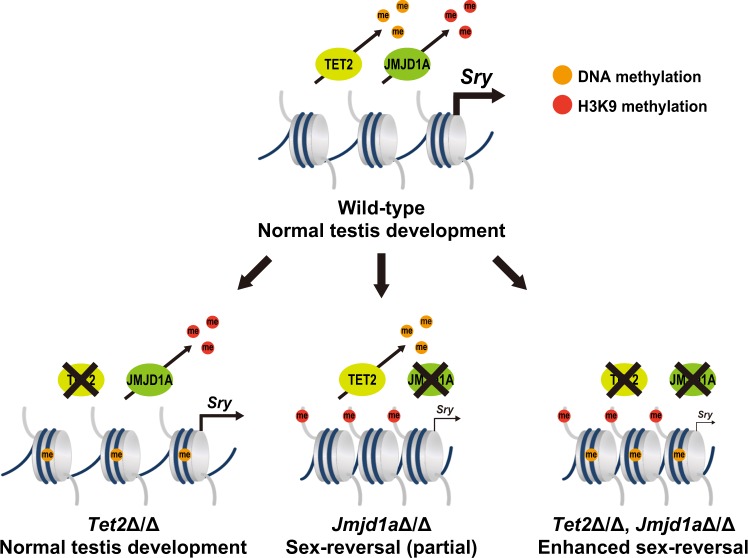
A schematic diagram for the regulation of *Sry* expression by TET2 and JMJD1A. Epigenetic regulation of *Sry* expression through H3K9 demethylation and DNA demethylation in male gonad development. TET2 catalyzes DNA demethylation in the *Sry* promoter. It is conceivable that *Sry* expression levels in *Tet2*-deficient gonads might be higher than the threshold required for testis development. JMJD1A-mediated H3K9 demethylation plays a dominant role in *Sry* activation. *Jmjd1a* deficiency lead to substantial reduction of *Sry* expression, thereby inducing sex-reversal. We found that *Tet2* deficiency synergistically enhanced the sex reversal phenotype of *Jmjd1a*Δ/Δ embryos.

Our previous results have shown that *Sry* mRNA is not completely abolished in XY *Jmjd1a*-deficient embryos. We therefore speculated that along with H3K9 demethylation, other epigenetic regulatory mechanism might also control *Sry* expression^10^. Our present study revealed that *Sry* activation is ensured by active DNA demethylation as well as by H3K9 demethylation (Fig. 5). In this model, JMJD1A-mediated H3K9 demethylation substantially contributes to *Sry* activation whereas TET2-mediated DNA demethylation acts supportively. *Tet2* deficiency leads to reduced *Sry* expression levels, whereas subsequent gonadal sex development proceeds normally. These facts strongly suggest that *Sry* expression levels in *Tet2*-deficient gonadal somatic cells are still higher than the threshold required for triggering the testis formation pathway^3^. In contrast, we found that *Tet2* deficiency synergistically enhances the sex reversal phenotype of *Jmjd1a*-deficient mice, indicating that TET2-mediated DNA demethylation practically contributes to mouse sex development.

Although *Tet1* and *Tet3* were expressed in gonadal somatic cells at the sex-determining period, DNA methylation at the *Sry* promoter was not affected by *Tet1* or *Tet3* deficiency (Fig. 3a,b). These results suggest that there might be an unrevealed mechanism by which *Sry* is demethylated specifically by TET2. Interestingly, the protein structure of TET2 differs from those of TET1 and TET3, as TET1/TET3, but not TET2, contain the CxxC domain that is essential for CpG site recognition. Therefore, it is plausible that recruitment of TET2 to its target loci depends on its interacting factor^29^. A zinc finger-type transcription factor WT1 plays an essential role in male sex-determination^30^. *In vitro* analysis has shown that WT1 (-KTS), one of the WT1 protein isoforms, can bind and activate *Sry* promoter^31–36^. Another study reported that WT1 directly binds to TET2, but not TET1, and recruits TET2 to the target genes^37^. In addition, WT1 displays high affinity for sequences containing 5mC rather than 5hmC or 5fC^38^. Considering these previous reports, it is possible that a specific DNA binding molecule mediates the recruitment of TET2 to the *Sry* promoter in gonadal somatic cells, and WT1 may be a promising candidate. We found that *Wt1* mRNA was constantly expressed in the developing XY gonads (Supplementary Fig. S4).

Although we showed that TET2 catalyzes active DNA demethylation at *Sry* promoter in gonadal somatic cells at the sex-determining period (Fig. 3c), we could not rule out the possibility that the TET protein-independent pathway also contributes to DNA demethylation at *Sry* promoter. For example, activation-induced deaminase (AID)/APOBEC-family cytosine deaminases are known to be involved in another pathway of active DNA demethylation. AID/APOBEC-family enzymes do not produce 5hmC but produce 5-methyluracil by deaminating the cytidine of 5mC^39^, which eventually converts to unmodified cytosine through the BER pathway^40,41^. Several studies have shown that APOBEC3 can discriminate against 5mC^39,42^. In fact, our mRNA expression analysis has demonstrated higher expression of *Apobec3* in gonadal somatic cells than that in mesonephric cells during gonadal development (Supplementary Fig. S3). Therefore, it is worth investigating whether the APOBEC3-mediated DNA demethylation pathway also plays a role in active DNA demethylation at the *Sry* promoter.

*Sry* demethylation is the earliest event in male embryonic gonad development^20^. Our data showed that the *Sry* promoter is demethylated during *Sry* induction through TET2-mediated active demethylation. Interestingly, we found that methylation of *Sry* promoter was maintained at low levels even at E12.0 (Fig. 2d), whereas *Sry* expression became diminished until this stage. These results were consistent with a previous report^20^. mRNA expression analysis demonstrated constant expression of *Dnmt3b* and increased expression of *Dnmt1* and *Dnmt3a* in the developing XY gonads from E10.6 to E12.0 (Supplementary Fig. S4). Taking these results together, we postulate that DNA methylation/demethylation are required for *Sry* induction but not for its repression. Instead, *Sry* repression might be achieved independently of epigenetic regulation. Transient expression of SRY initiates *Sox9* expression^43^. Once SOX9 reaches a critical threshold, *Sry* is repressed by a SOX9-dependent negative-feedback loop^43,44^. It is conceivable that *Sry* repression might be achieved by such feedback loop of transcription factors.

Other than JMJD1A and TET2, we previously demonstrated that an H3K9 methyltransferase complex, GLP/G9a is involved in *Sry* regulation with an antagonistic function against JMJD1A^28^. Moreover, recent studies have revealed that histone acetyltransferases p300/CBP play a crucial role in *Sry* activation^11^ and that polycomb-group protein CBX2 is involved in testis development by repressing Wnt signal^45^. These findings indicate that multiple epigenetic modifiers contribute to the complicated process of gonadal sex differentiation in mammals. However, it is still unclear and further study is required to determine how these enzymes are recruited to specific target loci including *Sry*, during gonadal sex development.

## Methods

### Animals

All animal experiments and methods were approved and performed in accordance with the relevant guidelines and regulations of the Animal Care Committee of Tokushima University (T29-62) and Osaka University (FBS-18-014). Mice (C57BL/6J and ICR) were supplied by SLC (Shimizu Laboratory Supplier, Kyoto) in Japan. Mouse lines of *Jmjd1a*-deficient mice and *Nr5a1-hCD271*-transgenic mice^10^ were sequentially backcrossed with C57BL/6J, and the F5 or later generation was used. Embryos were staged by counting tail somites as described, where approximately E10.6, E11.4, and E12.0 correspond to 9–10 ts, 16–17 ts, and 22–23 ts, respectively.

### Generation of *Tet* mutant mice using a CRISPR/Cas9 system

Mutant mice for each *Tet* (*Tet1*/*2*/*3*) were produced by electroporating *Cas9* mRNA and gRNA into mouse zygotes according to the protocol published recently^46^. Briefly, 400 ng/µl *Cas9* mRNA and 100 ng/µl each gRNAs targeting *Tets* (shown in Supplementary Fig. S2) were introduced into zygotes (C57BL/6 J × C57BL/6 J) by electroporation using Genome Editor GEB15 (BEX, Tokyo, Japan). The electroporation conditions were 30 V (3 msec ON + 97 msec OFF) × 4 times. The surviving 2-cell-stage embryos were transferred to the oviducts of pseudopregnant females (ICR). Mutant mice were backcrossed for more than three generations into the C57BL6/J background.

### Magnetic-activated cell sorting (MACS)

MACS was performed as described previously^18^. Briefly, a single cell suspension was prepared by trypsinizing the gonads and mesonephros isolated from embryos carrying the *Nr5a1/CD271*-transgene. Immunomagnetic isolation of CD271-expressing cells was performed according to the standard protocol (Miltenyi Biotech).

### RNA expression analysis

Total RNA from hCD271-positive/-negative cells and a pair of gonads/mesonephros was extracted using Trizol (Invitrogen). ReverTra Ace qPCR RT Kit (TOYOBO) was used for cDNA synthesis, following the manufacturer’s instructions. Subsequently, cDNA was used as the template for RT-qPCR using a StepOnePlus Real-Time PCR System (Applied Biosystems) and SYBR Premix Ex Taq II (TaKaRa) with gene-specific primers. The primer sets used in this analysis were as follows:

Sry forward 5′-TACCTACTTACTAACAGCTGACATCAC-3′

Sry reverse 5′-TGTCATGAGACTGCCAACCACAGGG-3′

Tet1 forward 5′-CCATTCTCACAAGGACATTCACA-3′

Tet1 reverse 5′-GCAGGACGTGGAGTTGTTCA-3′

Tet2 forward 5′-GCCATTCTCAGGAGTCACTGC-3′

Tet2 reverse 5′-CTTCTCGATTGTCTTCTCTATTGAGG-3′

Tet3 forward 5′-TCAGGGATGCTTTCTGTAGGG-3′

Tet3 reverse 5′-ATTTGGGATGTCCAGATGAGC-3′

Apobec3 forward 5′-AGCATGCAGAAATCCTCTTCC-3′

Apobec3 reverse 5′-AGATCTGGACGATCCCTTTTG-3′

Dnmt1 forward 5′-ATCAGGTGTCAGAGCCCAAAG-3′

Dnmt1 reverse 5′-TGGTGGAATCCTTCCGATAAC-3′

Dnmt3a forward 5′- CAGACGGGCAGCTATTTACAG-3′

Dnmt3a reverse 5′- TGGTTCTCTTCCACAGCATTC-3′

Dnmt3b forward 5′-TCGCAAGGTGTGGGCTTTTGT-3′

Dnmt3b reverse 5′-CTGGGCATCTGTCATCTTTGCA-3′

Wt1 forward 5′-ATGGCCACACGCCCTCGCATCACGC-3′

Wt1 reverse 5′-AGCGAGCCCTGCTGGCCCATGGGGT-3′

Gapdh forward 5′-ATGAATACGGCTACAGCAACAGG-3′

Gapdh reverse 5′-CTCTTGCTCAGTGTCCTTGCTG-3′

### Bisulfite sequencing analysis

Total genomic DNA was isolated from hCD271-positive/-negative gonad-mesonephros pairs of 3 embryos or from 3 whole embryos (E8.5). Genomic DNA was extracted using the Wizard SV Genomic DNA Purification System (Promega). Genomic DNA was treated with sodium bisulfite using the MethylEasy Xceed Rapid DNA Bisulfite Modification Kit (Human Genetic Signatures) following the manufacturer’s instructions. The bisulfite-treated DNA was PCR-amplified using respective primer sets. PCR products were subcloned into the pGEM-T Easy vector (Promega) and sequenced. Methylation levels of *Sry* promoter analyzed by every >30 sequencing. The primer sets used in this analysis were as follows:

Sry 1st forward 5′-TTTATATTGGGTTATAGAGTTAGAATAGAT-3′

Sry 2nd forward 5′-AGATTATTTTTTAGTTTTTTTTATGT-3′

Sry 1st and 2nd reverse 5′-CCAAAATATACTTATAACAAAAATTTTAAT-3′

Sox9 1st forward 5′-GTAGTAGAAGTTTTAGTTATTAT-3′

Sox9 2nd forward 5′-GATTATTTTTAAGTATTTTTTTT-3′

Sox9 1st and 2nd reverse 5′-ATATAAATTTACTCTCTACTCTC-3′

Foxl2 1st forward 5′-GGTTTTGTTTTTTTTTATTTGAA-3′

Foxl2 2nd forward 5′-GAGGTTTGGATTATTTTTTTTT-3′

Foxl2 1st and 2nd reverse 5′-ATTTTCTTAACTAAACTCTCCC-3′

### Tet-assisted bisulfite sequencing analysis

The 5hmC TAB-seq kit (WiseGene) was used for converting 5mC to 5caC, according to the manufacturer’s instructions. Briefly, 5hmC in genomic DNA was first protected with glycosylation by β-glycosyltransferase for 6 h at 37 °C. Genomic DNA was then treated with Tet protein overnight at 37 °C. Tet oxidized DNA was treated with sodium bisulfite using the same method as in bisulfite sequencing analysis. For verification, 5mC control DNA and 5hmC control DNA were mixed at 0.5% and 0.3% volume of total gDNA, respectively.

### Immunohistochemical staining

This method was performed as described previously^26^. Briefly, tissues were fixed in 4% paraformaldehyde, embedded in Tissue-Tek OTC compound (Sakura Finetek Japan) and cut into 10-μm sections. The sections were incubated with primary antibodies (rat anti-Nr5a1 (Trans Genic Lnc, KO610), rabbit anti-5hmC (Active Motif, #39791), anti-Sox9 (Millipore, AB5535), goat anti-Foxl2 (Abcam, ab-5096)), overnight at 4 °C. For fluorescence staining, the sections were incubated with Alexa-conjugated secondary antibodies (Life Technologies) at room temperature for 1 h and counterstained with DAPI. The sections were mounted in Vectashield (Vector) and observed with a confocal laser scanning microscope (LSM700, Carl Zeiss). Co-immunostaining for Nr5a1 and 5hmC was performed in the order: antibody reaction for Nr5a1 (1st: 4 °C, O/N and 2nd: RT, 1 h) → 2 N HCl treatment (RT, 20 min) → antibody reaction for 5hmC (1st: RT, 1 h and 2nd: RT, 1 h).

### *In vivo* inhibitor analysis

3-AB (Sigma) was prepared as a 2.5 g/ml stock solution with DMSO; 4 ml of the 3-AB stock solution was diluted with 500 ml of PBS^47^. Pregnant females at E10.5 were intraperitoneally administered 500 ml of the inhibitor solution.

### Dot-blot analysis

Total genomic DNA was isolated from hCD271-positive/-negative cells of the gonad-mesonephros pairs of 3 embryos. Sonicated genomic DNA was prepared as 25, 50, 100 ng/µl. 1 µl of sample was spotted onto the nitrocellulose membrane. After baking at 80 °C for 1 h and blocking with 5% non-fat milk PBS-T for 1 h at room temperature, the membrane was incubated overnight with anti-5hmC antibody (Active Motif, #39791, 1:10,000) at 4 °C. Following the primary antibody reaction, the membrane was incubated with secondary horseradish peroxidase-coupled antibody. Detection was performed using the Western Lighting Plus-ECL (PerkinElmer). To ensure equal loading, the membrane was stained with methylene blue post-immunoblotting.

### RNA-seq analysis

Total RNA from hCD271-positive cells of 3 embryos was extracted using the Direct-zol RNA MicroPrep kit (ZYMO RESEACH). Quality control check for length distribution and concentration of RNA fragments in the prepared libraries was performed using the Agilent 2100 Bioanalyzer (Agilent Technologies). The cDNA library was constructed using standard methods (Illumina TruSeq mRNA stranded kit) with index adapters (Illumina TruSeq indexes). DNA was sequenced as single-end, 50 base-length reads on the Illumina HiSeq. 1500 instrument (Illumina Inc.) with 10 million reads. Raw sequence reads were mapped to mm10 by STAR (v2.6.0a) with mostly default parameters except the following:–outFilterMultimapNmax 1. TPM (Transcripts Per Kilobase Million) for *Sry*, *Tet1*, *Tet2*, and *Tet3* was estimated using the rsem-calculate-expression command of RSEM (v1.3.1). The TPM values for all genes are listed in Supplementary Table 1. RNA sequencing data have been uploaded to NCBI Sequence Read Archive (SRA) with the accession number PRJNA557299.

## Supplementary information

Supplementary Table 1

Supplementary Figures
