# Supplementary material for: TET2 catalyzes active DNA demethylation of the Sry promoter and enhances its expression
Source: Sci Rep. 2019 Sep 17;9:13462. doi: 10.1038/s41598-019-50058-7 (PMC6748950; doi:10.1038/s41598-019-50058-7)
Supplement: Supplementary file 2 — Supplementary Figures [file 41598_2019_50058_MOESM2_ESM.pdf]

TET2 catalyzes active DNA demethylation of the *Sry* promoter and enhances its expression

Naoki Okashita, Shunsuke Kuroki, Ryo Maeda, Makoto Tachibana

Supplementary Figure 1

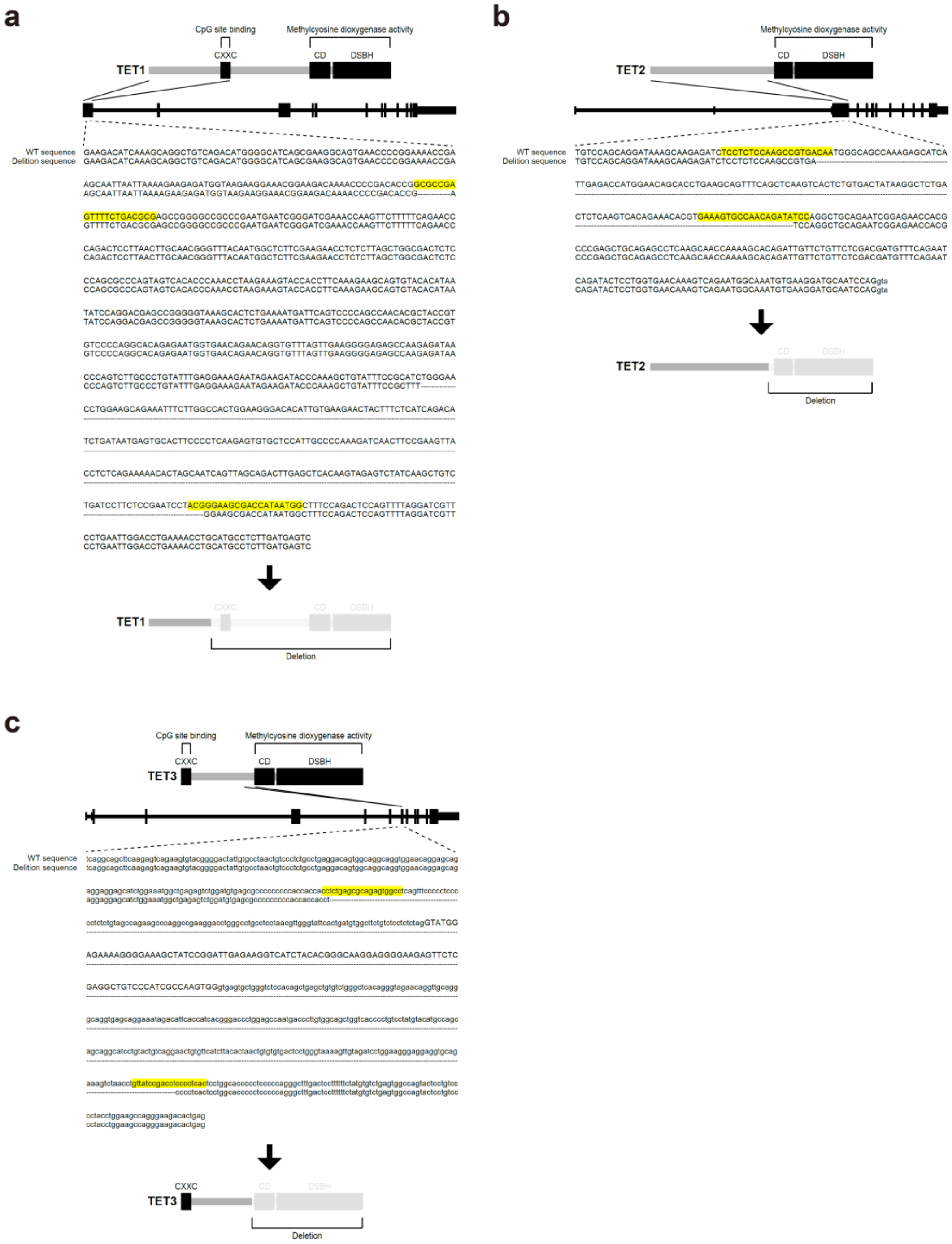

Supplementary Figure S1: Generation of *Tet1*/*Tet2*/*Tet3*-deficent mice by genome editing with the CRISPR/Cas9 system. (a-c) Protein and genomic structures of TET1/*Tet1* (a), TET2/*Tet2* (b), and TET3/*Tet3* (c) are represented at the top. We intended to disrupt the exon1 of *Tet1* (a) or the exon3 of *Tet2* (b) and the exon7 of *Tet3* (c) using two guide RNAs (gRNAs). Genomic sequences of wild-type and the corresponding mutant allele are shown at the bottom. The positions of the target sites of gRNAs are indicated in yellow. Dashed lines represent deleted sequences. Capital and lowercase letters represent exonic and intronic sequences, respectively. The final structure of each protein is shown at the bottom.

Supplementary Figure 2

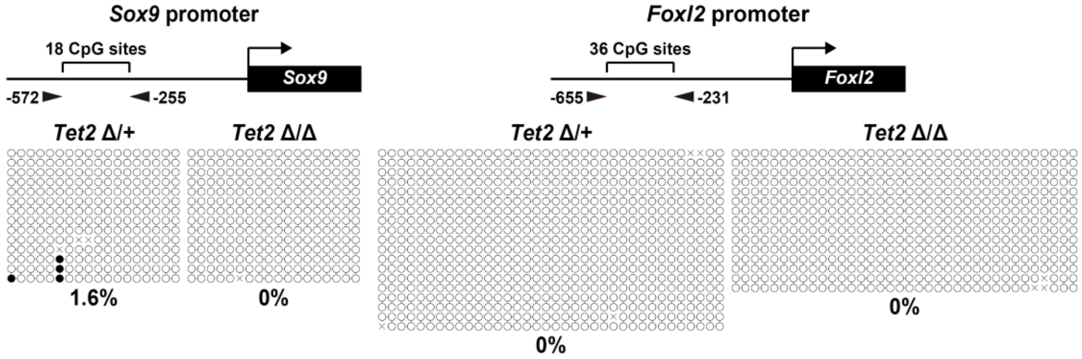

Supplementary Figure S2: DNA methylation levels in *Sox9 and Foxl2* promoters in E11.5 *Tet2* mutant gonadal somatic cells. Bisulfite sequencing analysis of promoters of *Sox9* (left) and *Foxl2* (right) in XY *Tet2*Δ/+ and *Tet2*Δ/Δ gonadal somatic cells at 16-17 ts stage. Schematic representation of the promoter is shown at the top. CpG sites are indicated relative to the start codon (upper). Arrows indicate primer positions. White circles indicate unmethylated cytosine, black circles indicate methylated cytosine and crosses indicate mutation of cytosine.

Supplementary Figure 3

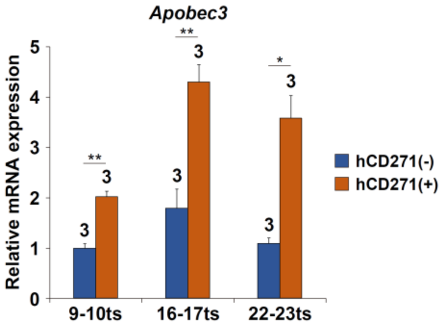

Supplementary Figure S3: Relative mRNA expression profile of *Apobec3*. qRT-PCR analysis of *Apobec3* in mesonephric and gonadal somatic cells at the indicated ts stages. mRNA expression levels in mesonephric cells at 9-10 ts were defined as 1. Data are presented as mean ± SD. * $P < 0.05$, ** $P < 0.01$. n = 3.

Supplementary Figure 4

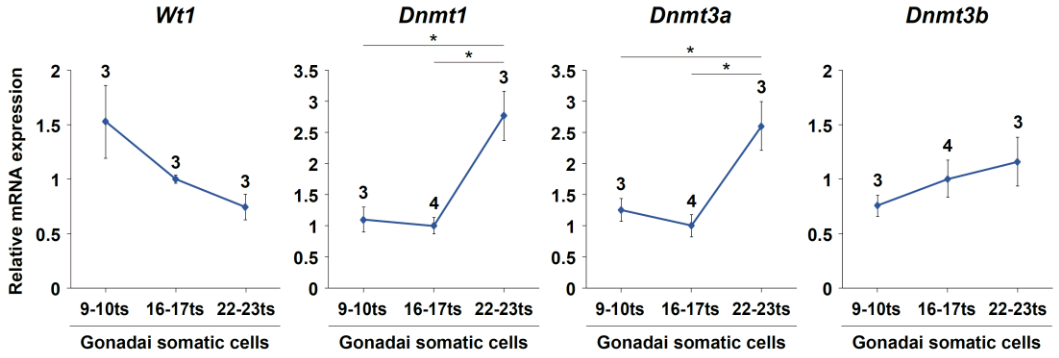

Supplementary Figure S4: Expression kinetics of *Dnmts* and *Wt1* in gonadal somatic cells. qRT-PCR analysis of *Dnmt1*, *Dnmt3a*, *Dnmt3b* and *Wt1* in XY gonadal somatic cells. mRNAs were collected from gonadal somatic cells at the indicated ts stages and introduced into qRT-PCR analysis. mRNA expression levels in gonadal somatic cells at 16-17 ts stage were defined as 1. Data are presented as mean ± SD. * $P < 0.05$, ** $P < 0.01$. n $\geq$ 3.
